# PAK4 signaling in health and disease: defining the PAK4–CREB axis

**DOI:** 10.1038/s12276-018-0204-0

**Published:** 2019-02-12

**Authors:** So-Yoon Won, Jung-Jin Park, Eun-Young Shin, Eung-Gook Kim

**Affiliations:** Department of Biochemistry, Chungbuk National University College of Medicine, Cheongju, 28644 Korea

**Keywords:** Cell signalling, Experimental models of disease, Cell death in the nervous system

## Abstract

p21-Activated kinase 4 (PAK4), a member of the PAK family, regulates a wide range of cellular functions, including cell adhesion, migration, proliferation, and survival. Dysregulation of its expression and activity thus contributes to the development of diverse pathological conditions. PAK4 plays a pivotal role in cancer progression by accelerating the epithelial–mesenchymal transition, invasion, and metastasis. Therefore, PAK4 is regarded as an attractive therapeutic target in diverse types of cancers, prompting the development of PAK4-specific inhibitors as anticancer drugs; however, these drugs have not yet been successful. PAK4 is essential for embryonic brain development and has a neuroprotective function. A long list of PAK4 effectors has been reported. Recently, the transcription factor CREB has emerged as a novel effector of PAK4. This finding has broad implications for the role of PAK4 in health and disease because CREB-mediated transcriptional reprogramming involves a wide range of genes. In this article, we review the PAK4 signaling pathways involved in prostate cancer, Parkinson’s disease, and melanogenesis, focusing in particular on the PAK4-CREB axis.

## Gene expression: The role of a regulatory enzyme in disease

An enzyme that regulates an important controller of gene expression may offer a therapeutic target for cancer and other diseases. cAMP response element-binding protein (CREB) interacts with various other proteins to switch a myriad of target genes on and off in different cells. A review by Eung-Gook Kim, Eun-Young Shin and colleagues at Chungbuk National University, Cheongju, South Korea, explores the interplay between CREB and an enzyme called p21-activated kinase 4 (PAK4) in human health and disease. PAK4, for example, has been shown to promote CREB’s gene-activating function in prostate cancer, and PAK4 overexpression is a feature of numerous other tumor types. Disruptions in PAK4-mediated regulation of CREB activity have also been observed in neurons affected by Parkinson’s disease. The authors see strong clinical promise in further exploring the biology of the PAK4-CREB pathway.

## Introduction

p21-Activated kinase (PAK) was initially identified as an effector of Rho GTPases that play a central role in reorganization of the cytoskeleton^1^. Early studies on this kinase thus focused on its signaling pathways that control cellular morphology, adhesion, and migration^2,3^. Later, its known roles expanded to a wide range of cellular functions, including cell proliferation and survival. The number of PAK family members has increased to six, and they are classified into group I (PAK1–3) and group II (PAK4–6) based on their structures and functions^4^.

**Fig. 1:**
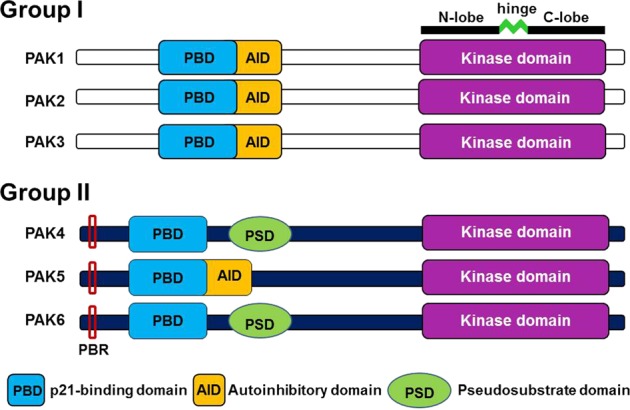
Domain architecture of PAK family kinases. Group I PAKs contain an overlapping PBD and AID in their N-terminal regions. Among the group II PAKs, PAK5 also contains a PBD and an AID. In contrast, PAK4 and PAK6 lack the AID but contain the PBD and PSD. Group II PAKs all contain a polybasic region (PBR), but its role has only been defined for PAK4 (see the main text for detail). N-lobe N-terminal lobe, C-lobe C-terminal lobe

In general, PAKs are composed of an N-terminal regulatory region and a C-terminal catalytic region (Fig. 1). Group I PAKs contain a p21-binding domain (PBD) and an autoinhibitory domain (AID) in the N-terminus, while group II PAKs contain a PBD and an AID or a pseudosubstrate domain (PSD), depending on the protein. The kinase domain of all PAK family members is located at the C-terminus. In the inactive state, group I PAKs are homodimers, and group II PAKs are monomers. The AID plays a key role in inhibiting kinase activity when group I PAKs are in the dimeric form. Upon binding of Rac/Cdc42 Rho GTPase to the PBD, AID-mediated inhibition is relieved, dissociating the dimer into monomers and thereby activating the kinase. However, controversy exists regarding whether the PBD in group II PAKs plays a similar role (Fig. 1). Group II PAKs show a binding preference for Cdc42 over Rac1. Binding of Cdc42 to the PBD of group II PAKs alters their intracellular location; for example, it can induce their translocation to the plasma membrane^5^. Moreover, a recent study revealed unexpected contact between Cdc42 and the polybasic region (PBR) and C-terminal lobe of PAK4 in addition to PBD^6^ (Fig. 1). These additional interactions were shown to suppress PAK4 kinase activity in vitro. Notably, PAK4 and PAK6 possess a PSD (Fig. 1), which blocks the entry of their substrates into the catalytic site; removal of this blockade by phosphorylation of S474 (human PAK4)/S602 (human PAK6) in the activation loop may represent an activation mechanism. Together with PSD-mediated inhibition, the extended Cdc42-PAK4 interactions may contribute to the full suppression of PAK4 kinase activity^6^.

**Fig. 2:**
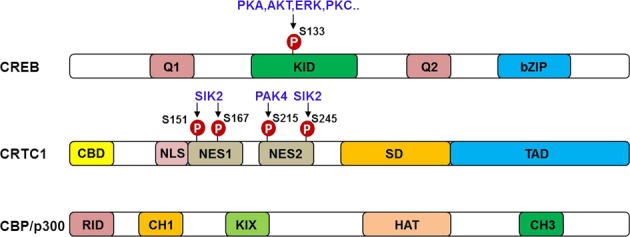
Domain architecture of CREB and its coactivators. The functional domains and major phosphorylation sites of CREB and its coactivators are shown. Kinases responsible for phosphorylating each amino acid residue are also depicted. KID kinase-inducible domain, CBD CREB-binding domain, NLS nuclear localization signal, NES nuclear export signal, SD splicing domain, TAD transactivation domain, RID nuclear receptor interaction domain, CH Cys- and His-rich region, KIX KID-interacting domain, HAT histone acetyltransferase

cAMP response element-binding protein (CREB) is a transcription factor that regulates the expression of a number of genes in diverse types of cells. Many signaling pathways converge on this factor, whose dysregulation subsequently leads to various pathological states, including carcinogenesis, abnormal metabolism, and neurodegeneration. Diverse posttranslational modifications contribute to regulation of the transcriptional activity of CREB. Phosphorylation of CREB has been extensively studied. Multiple kinases have been shown to directly phosphorylate CREB (Fig. 2): protein kinase A (PKA), protein kinase B (PKB/AKT), p42/44 mitogen-activated kinase (MAPK), and 90 kD ribosomal S6 kinase^7–10^. PKA is a heterotetramer composed of two regulatory subunits and two catalytic subunits. Four molecules of cAMP bind to the two regulatory subunits, resulting in the release of the catalytic subunits. Active free forms of the catalytic subunits phosphorylate CREB on S133, which induces its translocation to the nucleus and subsequent binding to CRE sites in the promoters of its target genes.

For its full activity, CREB requires cofactors such as CREB-binding proteins (CBPs) and CREB-regulated transcriptional cofactors (CRTCs) (Fig. 2). CREB phosphorylation induces the recruitment of CBPs, which enhance CREB activity; multiple phosphorylation sites in the kinase-inducible domain (KID) of CREB and the kinases that phosphorylate these sites have been documented^11^. In contrast, CREB phosphorylation is not required for its binding to CRTCs. Instead, CRTC dephosphorylation controls CREB activity^12^.

Recently, PAK4 has emerged as a novel regulator of CREB in health and disease. In this article, we will review PAK4 signaling, particularly in the PAK4–CREB axis, and discuss its clinical implications.

## PAK4 signaling pathways in cancer

**Fig. 3:**
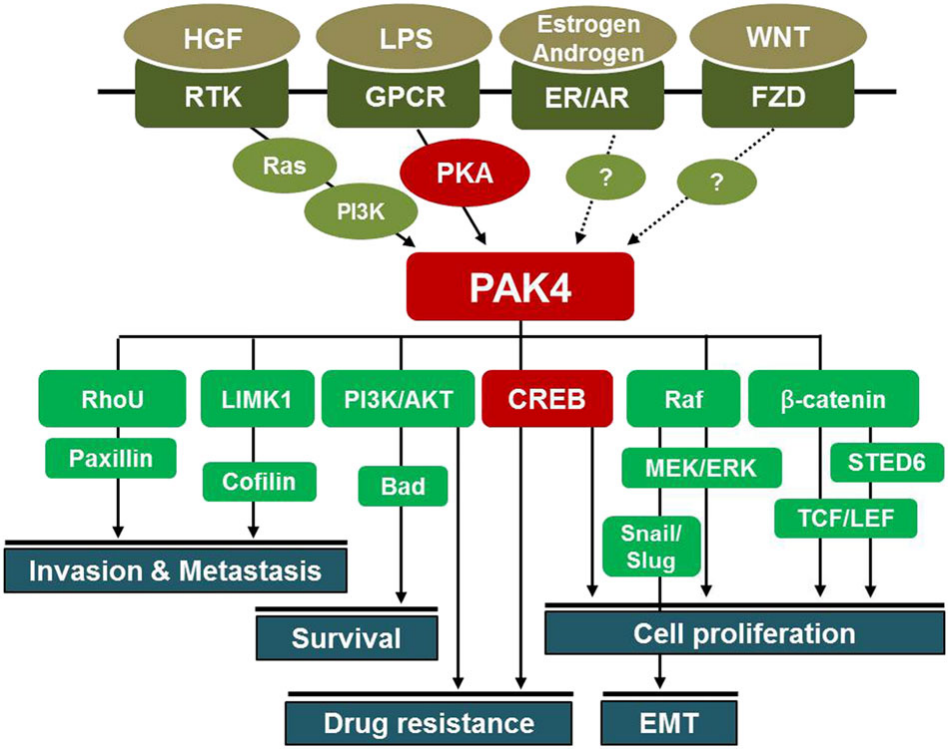
PAK4-mediated signaling pathways in cancer. PAK4 is activated by various extracellular signals through receptor tyrosine kinases (RTKs), G protein-coupled receptors (GPCRs), ERs, ARs, and WNT-FZD receptors. Activated PAK4 regulates cell proliferation, survival, invasion, metastasis, the epithelial-mesenchymal transition (EMT), and drug resistance in cancers (see the main text for a detailed description). In GPCR and RTK signaling, PKA and Ras, respectively, function upstream of PAK4. The upstream regulators of PAK4 in ER/AR and WNT signaling remain to be determined. HGF hepatocyte growth factor, LPS lipopolysaccharide

Deregulated proliferation, apoptosis, migration, adhesion, and invasion are hallmarks of cancer cells^13^. PAKs play important roles in these events; thus, unsurprisingly, their overexpression, gene amplification, and hyperactivation (although mutations leading to hyperactivation are rare) contribute to the development and progression of many cancers. A number of studies have revealed PAKs as hub molecules linking major signaling pathways, including the Ras–ERK, Wnt/β-catenin, and androgen receptor/estrogen receptor (AR/ER)-dependent pathways^14–18^ (Fig. 3). Because of this strategic role, PAKs, particularly PAK1 and PAK4, have emerged as attractive targets in the field of cancer therapy.

PAK4 is overexpressed in breast, gastric, prostate, lung, gallbladder, and ovarian cancers^19–24^. Amplification of the PAK4 gene, which is located on chromosome 19 (19q13.2)^25^, is frequently detected in ovarian, breast, and pancreatic cancer, as well as in squamous cell carcinoma^25–28^. PAK4 controls cell proliferation, survival, invasion, metastasis, the epithelial–mesenchymal transition (EMT), and drug resistance in vitro and in vivo (Fig. 3), thereby promoting overall cancer progression^14,21,22,29,30^. Notably, PAK4, but not the other PAK isoforms, is sufficient to transform normal cells^31^.

PAK4 is activated through diverse signaling pathways in cancer (Fig. 3). Oncogenic forms of Ras (mostly K-Ras) prevail in many human cancers, including pancreatic, colorectal, and lung adenocarcinomas^32–34^. Numerous approaches targeting mutant Ras have not yet been successful. Based on accumulating evidence, PAK4 is an alternative target in mutant K-Ras-driven cancers. PAK4 knockdown inhibits the proliferation of HCT116 colon cancer cells harboring mutant K-Ras^14^. This inhibition is independent of Raf/MEK/ERK or PI3K/AKT signaling, suggesting the involvement of unidentified PAK4 effectors. PAK4 activation mediates hepatocyte growth factor (HGF)-induced changes in the cytoskeleton and in cell adhesion downstream of PI3K;^15^ these changes require a physical interaction between PAK4 and the p85 alpha subunit of PI3K^35^. This interaction may also explain HGF-stimulated invasion in pancreatic ductal adenocarcinoma^35^, which frequently shows amplification of the PAK4 gene^25^. In contrast to the role of PAK4 as an effector of PI3K, PAK4 may act upstream of PI3K in promoting cisplatin resistance in gastric and cervical cancer cells^16,36^. A feedback loop between Ras/PI3K and PAK4 may explain these apparently incompatible findings. Development of specific inhibitors dissociating PI3K from PAK4 may represent a novel therapeutic modality in diverse types of cancers in which PI3K and PAK4 play a central role in progression^37^.

Aberrant activation of the canonical Wnt pathway is a hallmark of many human cancers, such as colorectal and hepatocellular carcinomas. PAK4 regulates the Wnt/β-catenin pathway through several mechanisms. PAK4 phosphorylates β-catenin at serine 675^38^, which is also phosphorylated by PAK1^17^. Phosphorylation at this site prevents β-catenin ubiquitination and subsequent proteasomal degradation in the cytoplasm. PAK4 is also involved in the nuclear transport of β-catenin^38^. A recent study revealed a mechanism underlying PAK4-mediated stabilization of β-catenin in the nucleus^38^. SETD6, a member of the family of protein lysine methyltransferases, methylates PAK4 bound to chromatin in cells^18^. This methylation induces close interaction between PAK4 and β-catenin, stabilization of β-catenin, and nuclear localization of β-catenin, resulting in increased β-catenin transcriptional activity^18^. Based on this evidence, PAK4 is a key regulator of the Wnt/β-catenin signaling pathway and thereby contributes to cancer progression. Researchers have not yet determined which Wnt ligands activate PAK4 and which activators function upstream of PAK4.

Early studies on PAK4 revealed its central role in actin cytoskeletal reorganization, which is similar to that of PAK1. HGF-activated PAK4 phosphorylates LIM kinase 1 (LIMK1)^39^, which phosphorylates and inactivates cofilin in migrating cells^40^, reducing the ability of cofilin to depolymerize F-actin. HGF is a potent agonist of tumor progression and invasiveness, and PAK4 is required for HGF-induced progression and invasion of human prostate cancer cells. The PAK4 gene is amplified in ~20% of patients with pancreatic cancer, and pancreatic tumors display increased PAK4 kinase activity^29^. The PAK4–LIMK1 pathway may contribute to the metastasis of pancreatic cancer. PAK4 is overexpressed in human non-small cell lung cancer (NSCLC), and its overexpression is associated with metastasis, decreased survival, and an advanced stage of NSCLC. PAK4 expression in NSCLC correlates with LIMK1 phosphorylation^22^. Overall, because of its importance in the reorganization of the actin cytoskeleton, the PAK4–LIMK1 pathway appears indispensable for the progression, including the invasion and metastasis, of prostate, lung, and pancreatic cancers.

PAK4 exerts its biological functions through both kinase-dependent and kinase-independent mechanisms^41,42^. Despite the presence of a PBD, PAK4 kinase activity appears to be independent of Rac/Cdc42 binding. What is the role of this PBD? Binding of Cdc42 to the PBD of PAK4 changes the subcellular localization of PAK4; for example, this binding can induce translocation of PAK4 to the plasma membrane^5^. Therefore, PAK4 may regulate cell adhesion and migration. PAK4 is expressed at high levels in breast cancer, with the highest levels detected in carcinomas with high grades and high invasiveness. PAK4 regulates the migration and adhesion turnover of these cells^42^. However, this regulatory pathway is independent of both its kinase activity and Cdc42 binding; surprisingly, binding of PAK4 to a nonconventional Rho GTPase, RhoU, regulates adhesion turnover^42^. This interaction stabilizes RhoU and prevents its ubiquitin-mediated destruction. Because of these kinase-dependent and kinase-independent modes of action, which are shared by other PAK isoforms^43,44^, inhibitors targeting the ATP-binding pockets of PAKs would be less effective therapeutic agents than similar inhibitors of other kinases.

### The PAK4–CREB axis in prostate cancer

The cAMP–PKA signaling pathway is a key regulator of tumorigenesis, tumor progression, chemotherapy resistance, and survival in patients with cancer; it is also a key regulator of survival, growth, and differentiation in normal cells^45^. CREB plays a central role in the cAMP signaling pathway by upregulating the expression of genes such as Bcl-2, Cyclin D1, and Egr-1^46^. CREB is overexpressed or hyperactivated in various human cancers, such as prostate cancer, NSCLC, brain tumors (glioblastoma), melanoma, acute leukemia, and breast cancer. Huang et al. identified CREB as a critical effector in prostate cancer bone metastasis^47^. In a recent study, we discovered that PAK4 regulates the transcriptional activity of CREB and thereby promotes prostate cancer progression through such mechanisms as the emergence of drug resistance and neuroendocrine differentiation^21^. The identification of GRK3 as a direct target of CREB supports a role for CREB in neuroendocrine differentiation^48^. PAK4-mediated CREB activation is independent of its phosphorylation on S133. In another study by our group, PAK4 was shown to activate CREB by phosphorylating CRTC1, a coactivator of CREB^49^. However, whether this mechanism is involved in tumorigenesis remains to be ascertained. Considering its critical role in cancer, many researchers have attempted to target CREB but have achieved limited success^45,50^. Strategies targeting the PAK4–CREB axis may represent an alternative therapeutic approach.

## PAK signaling in Parkinson’s disease

### Overview of Parkinson’s disease

Parkinson’s disease (PD) is a chronic, slowly progressing neurological disease^51^. PD is defined by the degeneration of dopaminergic neurons in the substantia nigra and the formation of Lewy body inclusions containing aggregated alpha-synuclein (α-Syn)^52^. The resulting dopamine deficiency in the basal ganglia leads to a movement disorder that is clinically characterized by parkinsonian motor symptoms such as bradykinesia, rest tremor, rigidity, postural instability, and gait impairment^53^. The etiology of PD remains unclear, but the disease may be caused by a combination of genetic and environmental factors^54,55^.

α-Syn has emerged as a critical protein in PD pathogenesis because its accumulation and aggregation have been mechanistically linked to PD pathogenesis^56,57^. According to accumulating evidence, aggregated α-Syn is the major toxic species that promotes cell death. Danzer et al. discovered that α-Syn oligomers, but not monomers, inhibit PAK4 kinase activity, as assessed by its autophosphorylation levels in vitro^58^. Furthermore, phosphorylation of the PAK4 substrate LIMK1 is reduced in brainstem extracts from α-Syn (A30P) transgenic mice^58^. Mutations in leucine-rich repeat kinase 2 (LRRK2) are the most common cause of monogenic PD^59^. LRRK2 mutations result in the accumulation of α-Syn aggregates and ubiquitin-positive inclusions in the brains of subjects with PD. Mitochondrial impairment results in ROS production in patients with PD^60,61^. Numerous studies have reported increases in the levels of several markers for oxidative damage in the substantia nigra of patients with PD^62^, indicating a contribution of impaired mitochondrial function to PD pathogenesis.

### PAK signaling pathways in Parkinson’s disease

Deregulation of PAK1 and PAK4 activity has been observed in subjects with PD^49,63^. Downregulation of PAK1 is involved in the loss of mesencephalic dopaminergic neurons^63^. Expression of a dominant-negative form of PAK1 (PAK1-DN) decreases neuronal cell viability and increases cell death induced by oxidative stress. PAK1-DN expression decreases levels of the Bcl-2 protein through a ubiquitin/proteasome-dependent mechanism^63^. Total levels of PAK4 and levels of active phosphorylated PAK4 are markedly reduced in patients with PD compared with age-matched controls^49^. Notably, PAK4 deficiency or inhibition renders dopamine neurons more vulnerable to 6-hydroxydopamine (6-OHDA)-mediated neurotoxicity in vivo^49^. Thus, deregulation of PAK1 and PAK4 is involved in PD pathogenesis and may provide potential therapeutic targets for the treatment of other neurodegenerative diseases.

### The PAK4–CREB axis in Parkinson’s disease

**Fig. 4:**
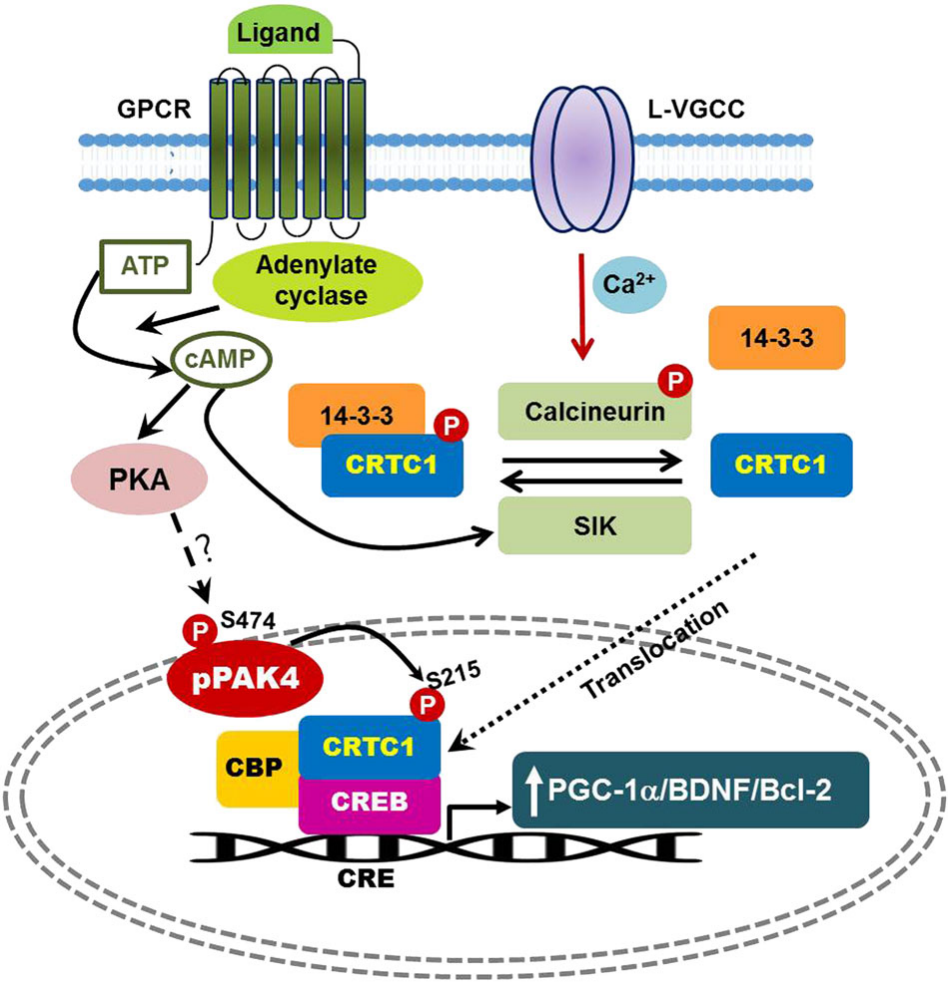
PAK4-mediated signaling pathways in PD. In the cytosol, CRTC1 is phosphorylated at S151 by the cAMP-SIK pathway and is inactivated through interactions with 14–3–3 proteins^12^. Ca^2 +^ influx through L-type VGCCs stimulates calcineurin-dependent CRTC1 dephosphorylation^12^. CRTC1 is activated by calcineurin-dependent dephosphorylation^12^. Dephosphorylated CRTC1 is translocated to the nucleus, where it is likely phosphorylated at S215 by PAK4, which triggers CREB-mediated transcription. In PD, oxidative stress and aggregated α-Syn decrease PAK4 activity and reduce pCRTC1^S215^ levels, which subsequently decrease the expression of CREB target genes such as BDNF, Bcl-2, and PGC-1α, leading to dopaminergic neuron death. CRE, cAMP response element; CREB CRE-binding protein, CRTC1 CREB-regulated transcription coactivator 1, CBP CREB-binding protein, SIK1 salt-inducible kinase 1, VGCC voltage-gated calcium channel, BDNF brain-derived neurotrophic factor, Bcl-2 B-cell lymphoma 2, PGC-1α peroxisome proliferator-activated receptor gamma coactivator 1 alpha, TH tyrosine hydroxylase

The expression of constitutively active PAK4 (caPAK4) protects dopaminergic neurons in both 6-OHDA and α-Syn rat models of PD and preserves motor function^49^. This neuroprotective effect of caPAK4 is mediated by the CREB transcription pathway (Fig. 4). CREB is well known to promote neuronal survival^64^. In the SH-SY5Y neuronal cell line, expression of caPAK4 increases CRE reporter activity and prevents 6-OHDA-induced suppression of the expression of the CREB target proteins PGC-1α, BDNF, and Bcl-2. CREB phosphorylation at S133 has been shown to be essential but not sufficient for CRE-driven gene transcription, suggesting the involvement of other mechanisms. The molecular mechanism by which PAK4 regulates CREB transcription does not require CREB phosphorylation (Fig. 4)^49^. A new family of CREB-specific coactivators has been identified, CRTC, which stands for “CREB-regulated transcription coactivator”^65^. The CRTC1 isoform is mainly expressed in the nuclei of dopaminergic neurons in the human and rat brain^49^. Cyclic AMP, salt-inducible kinase (SIK) and calcineurin antagonistically regulate CRTC-1-CREB signaling (Fig. 4). CRTC1 is inactive in the cytoplasm; cAMP and SIK phosphorylate CRTC1 and sequester it through interactions with 14–3–3 proteins. For CRTC1 activation, calcium influx through VGCCs stimulates calcineurin-dependent dephosphorylation of CRTC1, which induces its nuclear translocation (Fig. 4). Nuclear CRTC1 binds CREB, which induces the transcription of CREB target genes such as the PGC-1α and BDNF genes and protects cells from ischemia^66^. Deregulation of CRTC1-dependent CREB transcriptional activity is implicated in Alzheimer’s disease, Huntington’s disease, ischemia and disturbances in circadian clock activity^67^. Moreover, CRTC1-deficient mice show depression-related behaviors^68^. These mice display decreased levels of dopamine metabolites, suggesting that CRTC1 regulates dopamine metabolism in subjects with PD^68^. PAK4 directly interacts with CRTC1 and phosphorylates it at S215 (Fig. 4)^49^. Knockdown of CRTC1 in dopaminergic neurons compromises the ability of caPAK4 to protect these neurons from 6-OHDA toxicity in a rat model of PD. The nonphosphorylated form, CRTC1^S215A^, compromises the ability of caPAK4 to induce the expression of the CREB target proteins Bcl-2, BDNF, and PGC-1α^49^. Thus, phosphorylation of CRTC1 at S215 is essential for PAK4-mediated CREB activation and neuroprotection. Most neuromelanin-positive dopaminergic neurons in the normal aged human brain contain high levels of nuclear pCRTC1^S215^. In contrast, pCRTC1^S215^ levels in dopaminergic neurons are significantly lower in postmortem brain tissues from patients with PD. Based on these findings, the PAK4–CRTC1^S215^–CREB pathway is impaired in subjects with PD.

## PAK4 signaling in melanogenesis

### Overview of melanogenesis

Melanogenesis (synthesis of the pigment melanin) occurs in melanocytes, which are derived from the neural crest^69^. Two types of melanin, dark brown/black eumelanin and light red/yellow pheomelanin, are synthesized in the melanosomes of melanocytes. Although melanin has diverse functions, its protective role in the skin has been extensively studied; it minimizes the hazardous effects of UV radiation by absorbing UV and converting it into heat^70^. Excessive or defective melanogenesis not only causes diverse types of skin pigmentation disorders but also produces cosmetic issues. Melanogenesis is thus tightly regulated through multiple signaling pathways in epidermal and hair follicle melanocytes.

Human skin is easily exposed to UV irradiation, which induces DNA damage in keratinocytes. In response to DNA damage, the transcription factor p53 is stabilized and induces the transcription of multiple target genes required for melanogenesis, including the gene encoding proopiomelanocortin (POMC), the precursor of the pigmenting hormones α-melanocyte-stimulating hormone (α-MSH) and adrenocorticotropic hormone (ACTH)^71^. α-MSH is secreted from keratinocytes and binds to its receptor, melanocortin-1 receptor (MC1R), which is expressed on the surface of neighboring melanocytes. α-MSH-stimulated MC1R initiates a signaling cascade that includes the cAMP–PKA pathway, which activates CREB^72^. CREB stimulates the transcription of the microphthalmia-associated transcription factor (MITF), a master regulator of melanogenesis, which induces the expression of the melanogenic enzymes tyrosinase, TRP-1 and TRP-2^73^. Heterozygous mutations in MITF lead to Waardenburg syndrome IIA^74,75^, which manifests as abnormal pigmentation and deafness. In addition to pigmentation, MITF also regulates the proliferation and survival of melanocytes;^73^ thus, its deregulation is closely linked to melanomagenesis.

**Fig. 5:**
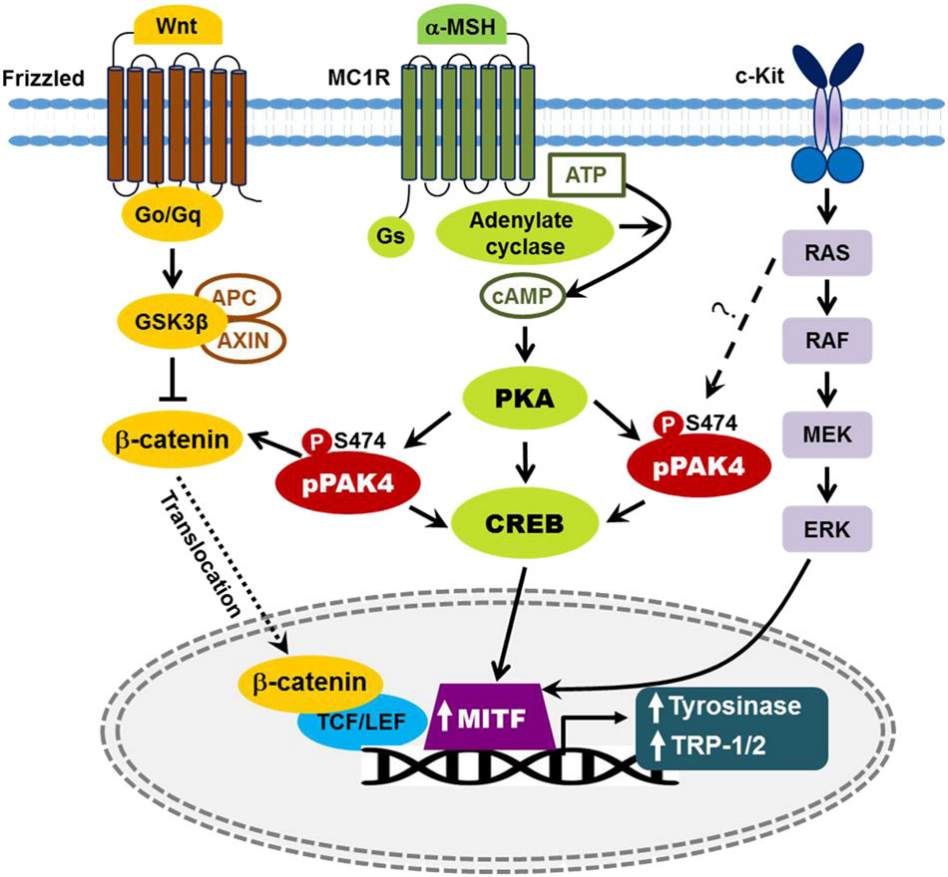
PAK4-mediated signaling pathways in melanogenesis. PAK4 is activated downstream of PKA in the α-MSH signaling pathway and activates the CREB–MITF pathway. PAK4 phosphorylates β-catenin and activates TCF/LEF-dependent transcription, but the Wnt ligands that activate PAK4 remain unclear. PAK4 also functions downstream of Ras, but the exact mechanism is unknown. MITF microphthalmia-associated transcription factor

The Wnt/β-catenin signaling pathway is essential for skin development through processes including the expansion of neural crest cells, the generation of melanoblasts, and the differentiation of melanoblasts into melanocytes^76,77^. Wnt/β-catenin signaling is also important for hair growth and wound healing^78,79^. Therefore, this signaling pathway has been the focus of extensive research in both epidermal and follicular stem cells^80,81^. Wnt signaling also plays a role in cutaneous pigmentation^81–84^. In the absence of Wnt ligands, β-catenin is subjected to ubiquitination-dependent proteolysis, and transcription of its downstream target MITF is subsequently inhibited. In the presence of Wnt ligands, binding to Frizzled and LRP5/6 coreceptors stabilizes β-catenin by disrupting the APC/Axin/GSK3β destruction complex, which increases MITF levels and stimulates melanogenesis. β-Catenin phosphorylation at S675 by PAK4 is another mechanism of β-catenin stabilization^38^. α-MSH induces the phosphorylation of β-catenin at S675^81^, but the mechanism has remained elusive. Our study revealed PAK4 as a downstream mediator of the effects of α-MSH^85^. We therefore postulate that α-MSH stabilizes β-catenin through phosphorylation at S675 by PAK4, which increases the coactivator activity of β-catenin and initiates TCF/LEF-dependent transcription of target genes, including MITF. Thus, PAK4 might enhance melanogenesis through crosstalk with the Wnt/β-catenin pathway. Overall, PAK4 is a central regulator of melanogenesis because it provides a signaling hub linking two major melanogenic pathways, the cAMP–PKA pathway and the Wnt/β-catenin pathway (Fig. 5).

Stem cell factor (SCF)/c-Kit (a tyrosine kinase receptor) signaling plays multiple roles in melanocytes, including roles in proliferation, differentiation, and hair shaft pigmentation. SCF was initially identified as a growth factor for melanocytes and mast cells^86^. Diverse types of skin cells, including keratinocytes, fibroblasts, and endothelial cells, secrete this factor. Similar to α-MSH, in response to UVB radiation, SCF is released from keratinocytes in a p53-dependent manner^87^. Mutations in the c-Kit gene are responsible for human piebaldism^88^, a rare autosomal pigmentation disorder caused by abnormal melanocyte development, which supports the importance of SCF/c-Kit signaling in melanocytes. SCF/c-Kit signaling activates the Ras/MAP kinase pathway, which leads to MITF phosphorylation^89^ and increases MITF transcriptional activity through recruitment of p300/CBP coactivators^90^. Upon stimulation by SCF, c-Kit is activated and recruits the guanine nucleotide exchange factor SOS and the adapter protein Grb2, which in turn activate Ras by converting Ras-GDP to Ras-GTP^91^. Active Ras then sequentially activates the RAF/MEK/ERK pathway. Interestingly, persistent activation of the BRAF/MEK/ERK MAP kinase (MAPK) pathway induces proteolytic degradation of MITF^92^, thereby inhibiting melanogenesis. This proteolytic step may involve ubiquitin-conjugating enzymes such as hUBC9^93^. MAP kinase increases CREB activity by phosphorylating CREB on S133; thus, SCF/c-Kit signaling upregulates melanogenesis via the pCREB^S133^–MITF pathway in addition to its direct effect on MITF phosphorylation. Furthermore, considering the role of PAK4 as a downstream effector of oncogenic Ras, we predict that PAK4 also functions downstream of Ras in SCF-stimulated melanocytes. These findings provide evidence for crosstalk between the α-MSH and SCF/c-Kit signaling pathways. Thus, melanogenesis is regulated by a complex array of signaling networks that involve the three major pathways described above and other pathways that are not reviewed here^94^.

### The PAK4–CREB axis in melanogenesis

We recently identified PAK4 as a downstream effector of PKA; PKA directly binds PAK4 and activates it by inducing the phosphorylation of S474^21^. This observation suggests a potential mechanism by which PAK4 regulates CREB activity globally in diverse cell types. Indeed, PAK4 increases the transcriptional activity of CREB in melanocytes^85^. As MITF is a downstream target of CREB, PAK4 activation enhances melanogenesis through the CREB–MITF pathway^85^. Initially, the mechanism by which PAK4 activates CREB was unclear because PAK4 does not directly phosphorylate CREB^21^. Dephosphorylation of CRTC was recently shown to facilitate its nuclear translocation and CREB activation, which occurs in a CREB phosphorylation-independent manner through recruitment of the transcriptional machinery^65,95^. SIK2 is a serine/threonine kinase that phosphorylates CRTC and thus inhibits its nuclear translocation and subsequent CREB activation. Indeed, depletion of SIK2, the predominant SIK isoform expressed in melanocytes, upregulates melanogenesis in mice^96^. A further study showing that small-molecule SIK inhibitors upregulate MITF and induce melanogenesis supports a critical role for CRTC in the regulation of the CREB–MITF pathway^97^. Interestingly, CRTC functions downstream of AMPK in adiponectin signaling to suppress UV radiation- and α-MSH-induced melanogenesis^98^. In contrast to a role for CRTC dephosphorylation as an activation mechanism, PAK4-mediated CRTC1 phosphorylation on S215 increased CREB activity through an unknown mechanism in our previous study^49^. According to a recent study, melanocytes express CRTC2 and CRTC3^98^. Consensus residues similar to those surrounding S215 in human CRTC1 are present in these isoforms (S244 in CRTC2 and S243 in CRTC3); thus, PAK4 likely regulates the CREB–MITF pathway by phosphorylating CRTC2 and CRTC3 in melanocytes.

Regarding the possible roles of other PAK isoforms in melanogenesis, we also detected PAK2 in melanocytes, but its knockdown did not affect melanogenesis^85^, suggesting that PAK2 is dispensable for this process. PAK1 is expressed in melanocytes at very low levels, but forced expression increases forskolin- and α-MSH-induced melanogenesis^99^. However, its function in the skin remains to be determined. PAK1 has been implicated in the development of melanoma^100^. PAK4 has been well documented as a tumor progression factor in a number of tumor types^101^, but its role in melanoma has not been studied. Deregulation of pigmentation genes is linked to the development of melanoma^102,103^. Because PAK4 plays a central role in melanogenesis, investigations into the mechanism by which deregulation of PAK4 contributes to the development of melanoma would be fruitful.

## Perspectives

In the current review, we have highlighted the role of PAK4 signaling in many biological events, including prostate cancer progression, neuroprotection in Parkinson’s disease, and the promotion of melanogenesis. Because CREB is a key transcription factor involved in diverse pathophysiologies and because PAK4 controls CREB activity, we postulate that PAK4 regulates many unidentified biological functions. For instance, the roles of CREB and CRTC2 in diabetes mellitus have been extensively studied^104,105^, suggesting the involvement of the PAK4-CRTC-CREB pathway in the pathogenesis of diabetes mellitus.

Regarding potential translational implications, the PAK4–CREB axis may represent a therapeutic target. Because of its frequent mutations in human cancers, numerous researchers have attempted to target mutated K-Ras^106^ but have not yet achieved success. Based on accumulating evidence, PAK4 represents an alternative target in mutant K-Ras-driven cancers. Recently, Karyopharm Therapeutics (Newton, MA, USA) has made notable progress by identifying PAK4 allosteric modulators and has provided proof of concept for the treatment of pancreatic cancer in animal models^107^. One of the identified compounds, KPT-9274, is currently being investigated in a phase I clinical trial.

Some questions remain unanswered. PKA regulates CREB activity by directly phosphorylating it on S133. Is the PAK4–CREB axis a safeguard for the biological functions of the cAMP signaling pathway? Our recent study revealed the Slug transcription factor as a direct target of PAK4 in the TGF-β signaling pathway^30^, suggesting a distinct role for PAK4 as a transcriptional regulator. In this regard, we are tempted to speculate that PAK4 activation downstream of PKA might partially explain the wide range of cellular functions of PKA reported in previous studies. Another interesting question is whether CRTC isoforms other than CRTC1 are regulated by PAK4 through similar mechanisms. Finally, researchers have not determined whether PAK5 or PAK6 functions downstream of PKA and then activates CREB.

## References

[1] Manser E, Leung T, Salihuddin H, Zhao ZS, Lim L (1994). A brain serine/threonine protein kinase activated by Cdc42 and Rac1. Nature.

[2] Nobes CD, Hall A (1999). Rho GTPases control polarity, protrusion, and adhesion during cell movement. J. Cell Biol..

[3] Manser E, Lim L (1999). Roles of PAK family kinases. Prog. Mol. Subcell. Biol..

[4] Arias-Romero LE, Chernoff J (2008). A tale of two Paks. Biol. Cell.

[5] Chenette EJ, Mitin NY, Der CJ (2006). Multiple sequence elements facilitate Chp Rho GTPase subcellular location, membrane association, and transforming activity. Mol. Biol. Cell.

[6] Ha BH, Boggon TJ (2018). CDC42 binds PAK4 via an extended GTPase-effector interface. Proc. Natl Acad. Sci. USA.

[7] Du K, Montminy M (1998). CREB is a regulatory target for the protein kinase Akt/PKB. J. Biol. Chem..

[8] Finkbeiner S (2001). New roles for introns: sites of combinatorial regulation of Ca2 + - and cyclic AMP-dependent gene transcription. Sci. STKE.

[9] Mayr B, Montminy M (2001). Transcriptional regulation by the phosphorylation-dependent factor CREB. Nat. Rev. Mol. Cell Biol..

[10] Wen AY, Sakamoto KM, Miller LS (2010). The role of the transcription factor CREB in immune function. J. Immunol..

[11] Shaywitz AJ, Greenberg ME (1999). CREB: a stimulus-induced transcription factor activated by a diverse array of extracellular signals. Annu. Rev. Biochem..

[12] Nonaka M (2014). Region-specific activation of CRTC1-CREB signaling mediates long-term fear memory. Neuron.

[13] Hanahan D, Weinberg RA (2011). Hallmarks of cancer: the next generation. Cell.

[14] Tabusa H, Brooks T, Massey AJ (2013). Knockdown of PAK4 or PAK1 inhibits the proliferation of mutant KRAS colon cancer cells independently of RAF/MEK/ERK and PI3K/AKT signaling. Mol. Cancer Res..

[15] Wells CM, Abo A, Ridley AJ (2002). PAK4 is activated via PI3K in HGF-stimulated epithelial cells. J. Cell Sci..

[16] Fu, X. et al. PAK4 confers cisplatin resistance in gastric cancer cells via PI3K/Akt- and MEK/ERK-dependent pathways. *Biosci. Rep.* **34**, e00094 (2014). 10.1042/BSR20130102 PMC3941610 27919028

[17] Zhu G (2012). A Rac1/PAK1 cascade controls beta-catenin activation in colon cancer cells. Oncogene.

[18] Vershinin Z, Feldman M, Chen A, Levy D (2016). PAK4 methylation by SETD6 promotes the activation of the Wnt/beta-catenin pathway. J. Biol. Chem..

[19] Wong LE, Chen N, Karantza V, Minden A (2013). The Pak4 protein kinase is required for oncogenic transformation of MDA-MB-231 breast cancer cells. Oncogenesis.

[20] Ahn HK (2011). P21-activated kinase 4 overexpression in metastatic gastric cancer patients. Transl. Oncol..

[21] Park MH (2013). p21-Activated kinase 4 promotes prostate cancer progression through CREB. Oncogene.

[22] Cai S (2015). Overexpression of P21-activated kinase 4 is associated with poor prognosis in non-small cell lung cancer and promotes migration and invasion. J. Exp. Clin. Cancer Res..

[23] Kim JH (2008). Gene expression profiles in gallbladder cancer: the close genetic similarity seen for early and advanced gallbladder cancers may explain the poor prognosis. Tumour Biol..

[24] Davis SJ (2015). Enhanced GAB2 expression is associated with improved survival in high-grade serous ovarian cancer and sensitivity to PI3K inhibition. Mol. Cancer Ther..

[25] Chen S (2008). Copy number alterations in pancreatic cancer identify recurrent PAK4 amplification. Cancer Biol. Ther..

[26] Davis SJ (2013). Functional analysis of genes in regions commonly amplified in high-grade serous and endometrioid ovarian cancer. Clin. Cancer Res..

[27] Yu W, Kanaan Y, Bae YK, Gabrielson E (2009). Chromosomal changes in aggressive breast cancers with basal-like features. Cancer Genet. Cytogenet..

[28] Begum A (2009). Identification of PAK4 as a putative target gene for amplification within 19q13.12-q13.2 in oral squamous-cell carcinoma. Cancer Sci..

[29] Yeo D, He H, Baldwin GS, Nikfarjam M (2015). The role of p21-activated kinases in pancreatic cancer. Pancreas.

[30] Park JJ (2018). The p21-activated kinase 4-Slug transcription factor axis promotes epithelial-mesenchymal transition and worsens prognosis in prostate cancer. Oncogene.

[31] Qu J (2001). Activated PAK4 regulates cell adhesion and anchorage-independent growth. Mol. Cell. Biol..

[32] Almoguera C (1988). Most human carcinomas of the exocrine pancreas contain mutant c-K-ras genes. Cell.

[33] Boughdady IS, Kinsella AR, Haboubi NY, Schofield PF (1992). K-ras gene mutations in adenomas and carcinomas of the colon. Surg. Oncol..

[34] Mills NE, Fishman CL, Rom WN, Dubin N, Jacobson DR (1995). Increased prevalence of K-ras oncogene mutations in lung adenocarcinoma. Cancer Res..

[35] King H (2017). PAK4 interacts with p85 alpha: implications for pancreatic cancer cell migration. Sci. Rep..

[36] Shu XR, Wu J, Sun H, Chi LQ, Wang JH (2015). PAK4 confers the malignance of cervical cancers and contributes to the cisplatin-resistance in cervical cancer cells via PI3K/AKT pathway. Diagn. Pathol..

[37] Thillai K, Lam H, Sarker D, Wells CM (2017). Deciphering the link between PI3K and PAK: an opportunity to target key pathways in pancreatic cancer?. Oncotarget.

[38] Li Y (2012). Nucleo-cytoplasmic shuttling of PAK4 modulates beta-catenin intracellular translocation and signaling. Biochim. Biophys. Acta.

[39] Dan C, Kelly A, Bernard O, Minden A (2001). Cytoskeletal changes regulated by the PAK4 serine/threonine kinase are mediated by LIM kinase 1 and cofilin. J. Biol. Chem..

[40] DesMarais V, Ghosh M, Eddy R, Condeelis J (2005). Cofilin takes the lead. J. Cell Sci..

[41] Gnesutta N, Minden A (2003). Death receptor-induced activation of initiator caspase 8 is antagonized by serine/threonine kinase PAK4. Mol. Cell. Biol..

[42] Dart AE (2015). PAK4 promotes kinase-independent stabilization of RhoU to modulate cell adhesion. J. Cell Biol..

[43] Galisteo ML, Chernoff J, Su YC, Skolnik EY, Schlessinger J (1996). The adaptor protein Nck links receptor tyrosine kinases with the serine-threonine kinase Pak1. J. Biol. Chem..

[44] Lu W, Katz S, Gupta R, Mayer BJ (1997). Activation of Pak by membrane localization mediated by an SH3 domain from the adaptor protein Nck. Curr. Biol..

[45] Steven A, Seliger B (2016). Control of CREB expression in tumors: from molecular mechanisms and signal transduction pathways to therapeutic target. Oncotarget.

[46] Sakamoto KM, Frank DA (2009). CREB in the pathophysiology of cancer: implications for targeting transcription factors for cancer therapy. Clin. Cancer Res..

[47] Huang WC (2006). beta2-microglobulin is a signaling and growth-promoting factor for human prostate cancer bone metastasis. Cancer Res..

[48] Sang M (2016). GRK3 is a direct target of CREB activation and regulates neuroendocrine differentiation of prostate cancer cells. Oncotarget.

[49] Won SY (2016). Nigral dopaminergic PAK4 prevents neurodegeneration in rat models of Parkinson’s disease. Sci. Transl. Med..

[50] Xiao X, Li BX, Mitton B, Ikeda A, Sakamoto KM (2010). Targeting CREB for cancer therapy: friend or foe. Curr. Cancer Drug Targets.

[51] Grayson M (2010). Parkinson’s disease. Nature.

[52] Sulzer D (2017). T cells from patients with Parkinson’s disease recognize alpha-synuclein peptides. Nature.

[53] Kravitz AV (2010). Regulation of parkinsonian motor behaviours by optogenetic control of basal ganglia circuitry. Nature.

[54] Tanner CM (2003). Is the cause of Parkinson’s disease environmental or hereditary? Evidence from twin studies. Adv. Neurol..

[55] Warner TT, Schapira AH (2003). Genetic and environmental factors in the cause of Parkinson’s disease. Ann. Neurol..

[56] Recasens A, Dehay B (2014). Alpha-synuclein spreading in Parkinson’s disease. Front. Neuroanat..

[57] Lazaro DF (2014). Systematic comparison of the effects of alpha-synuclein mutations on its oligomerization and aggregation. PLoS Genet..

[58] Danzer KM, Schnack C, Sutcliffe A, Hengerer B, Gillardon F (2007). Functional protein kinase arrays reveal inhibition of p-21-activated kinase 4 by alpha-synuclein oligomers. J. Neurochem..

[59] Martin I, Kim JW, Dawson VL, Dawson TM (2014). LRRK2 pathobiology in Parkinson’s disease. J. Neurochem..

[60] Bose A, Beal MF (2016). Mitochondrial dysfunction in Parkinson’s disease. J. Neurochem..

[61] Zorov DB, Juhaszova M, Sollott SJ (2014). Mitochondrial reactive oxygen species (ROS) and ROS-induced ROS release. Physiol. Rev..

[62] Dias V, Junn E, Mouradian MM (2013). The role of oxidative stress in Parkinson’s disease. J. Parkinsons Dis..

[63] Kim H (2016). Down-regulation of p21-activated serine/threonine kinase 1 is involved in loss of mesencephalic dopamine neurons. Mol. Brain.

[64] Mantamadiotis T (2002). Disruption of CREB function in brain leads to neurodegeneration. Nat. Genet..

[65] Altarejos JY, Montminy M (2011). CREB and the CRTC co-activators: sensors for hormonal and metabolic signals. Nat. Rev. Mol. Cell Biol..

[66] Sasaki T (2011). SIK2 is a key regulator for neuronal survival after ischemia via TORC1-CREB. Neuron.

[67] Xue ZC, Wang C, Wang QW, Zhang JF (2015). CREB-regulated transcription coactivator 1: important roles in neurodegenerative disorders. Sheng Li Xue Bao.

[68] Meylan EM, Halfon O, Magistretti PJ, Cardinaux JR (2016). The HDAC inhibitor SAHA improves depressive-like behavior of CRTC1-deficient mice: possible relevance for treatment-resistant depression. Neuropharmacology.

[69] Bonaventure J, Domingues MJ, Larue L (2013). Cellular and molecular mechanisms controlling the migration of melanocytes and melanoma cells. Pigment Cell Melanoma Res..

[70] Brenner M, Hearing VJ (2008). The protective role of melanin against UV damage in human skin. Photochem. Photobiol..

[71] Cui R (2007). Central role of p53 in the suntan response and pathologic hyperpigmentation. Cell.

[72] Garcia-Borron JC, Abdel-Malek Z, Jimenez-Cervantes C (2014). MC1R, the cAMP pathway, and the response to solar UV: extending the horizon beyond pigmentation. Pigment Cell Melanoma Res..

[73] Levy C, Khaled M, Fisher DE (2006). MITF: master regulator of melanocyte development and melanoma oncogene. Trends Mol. Med..

[74] Hughes AE, Newton VE, Liu XZ, Read AP (1994). A gene for Waardenburg syndrome type 2 maps close to the human homologue of the microphthalmia gene at chromosome 3p12-p14.1. Nat. Genet..

[75] Tassabehji M (1994). PAX3 gene structure and mutations: close analogies between Waardenburg syndrome and the Splotch mouse. Hum. Mol. Genet..

[76] Lim, X. & Nusse, R. Wnt signaling in skin development, homeostasis, and disease. *Cold Spring Harb. Perspect. Biol.* **5**, a008029 (2013). 10.1101/cshperspect.a008029 PMC3552514 23209129

[77] Ikeya M, Takada S (1998). Wnt signaling from the dorsal neural tube is required for the formation of the medial dermomyotome. Development.

[78] Shi Y (2015). Wnt and Notch signaling pathway involved in wound healing by targeting c-Myc and Hes1 separately. Stem Cell Res. Ther..

[79] Han L (2018). Activation of Wnt/beta-catenin signaling is involved in hair growth-promoting effect of 655-nm red light and LED in in vitro culture model. Lasers Med. Sci..

[80] Choi YS (2013). Distinct functions for Wnt/beta-catenin in hair follicle stem cell proliferation and survival and interfollicular epidermal homeostasis. Cell Stem Cell.

[81] Bellei B, Pitisci A, Catricala C, Larue L, Picardo M (2011). Wnt/beta-catenin signaling is stimulated by alpha-melanocyte-stimulating hormone in melanoma and melanocyte cells: implication in cell differentiation. Pigment Cell Melanoma Res..

[82] Dorsky RI, Raible DW, Moon RT (2000). Direct regulation of nacre, a zebrafish MITF homolog required for pigment cell formation, by the Wnt pathway. Genes Dev..

[83] Kim JY, Lee TR, Lee AY (2013). Reduced WIF-1 expression stimulates skin hyperpigmentation in patients with melasma. J. Invest. Dermatol..

[84] Yamada T (2013). Wnt/beta-catenin and kit signaling sequentially regulate melanocyte stem cell differentiation in UVB-induced epidermal pigmentation. J. Invest. Dermatol..

[85] Yun CY (2015). p21-activated kinase 4 critically regulates melanogenesis via activation of the CREB/MITF and beta-catenin/MITF pathways. J. Invest. Dermatol..

[86] Grabbe J (1994). Comparative cytokine release from human monocytes, monocyte-derived immature mast cells, and a human mast cell line (HMC-1). J. Invest. Dermatol..

[87] Hachiya A (2004). Biphasic expression of two paracrine melanogenic cytokines, stem cell factor and endothelin-1, in ultraviolet B-induced human melanogenesis. Am. J. Pathol..

[88] Wen GD (2013). A novel mutation of the KIT gene in a Chinese family with piebaldism. Chin. Med. J..

[89] Hemesath TJ, Price ER, Takemoto C, Badalian T, Fisher DE (1998). MAP kinase links the transcription factor Microphthalmia to c-Kit signalling in melanocytes. Nature.

[90] Price ER (1998). Lineage-specific signaling in melanocytes. C-kit stimulation recruits p300/CBP to microphthalmia. J. Biol. Chem..

[91] Thommes K, Lennartsson J, Carlberg M, Ronnstrand L (1999). Identification of Tyr-703 and Tyr-936 as the primary association sites for Grb2 and Grb7 in the c-Kit/stem cell factor receptor. Biochem. J..

[92] Wu M (2000). c-Kit triggers dual phosphorylations, which couple activation and degradation of the essential melanocyte factor Mi. Genes Dev..

[93] Xu W (2000). Regulation of microphthalmia-associated transcription factor MITF protein levels by association with the ubiquitin-conjugating enzyme hUBC9. Exp. Cell Res..

[94] Pillaiyar T, Manickam M, Jung SH (2017). Recent development of signaling pathways inhibitors of melanogenesis. Cell. Signal..

[95] Gu Y (2012). Altered LKB1/CREB-regulated transcription co-activator (CRTC) signaling axis promotes esophageal cancer cell migration and invasion. Oncogene.

[96] Horike N (2010). Downregulation of SIK2 expression promotes the melanogenic program in mice. Pigment Cell Melanoma Res..

[97] Mujahid N (2017). A UV-independent topical small-molecule approach for melanin production in human skin. Cell Rep..

[98] Bang S (2017). Novel regulation of melanogenesis by adiponectin via the AMPK/CRTC pathway. Pigment Cell Melanoma Res..

[99] Be TuPT (2017). The serum/PDGF-dependent “melanogenic” role of the minute level of the oncogenic kinase PAK1 in melanoma cells proven by the highly sensitive kinase assay. Drug Discov. Ther..

[100] Taira N, Nguyen BC, Be Tu PT, Tawata S (2016). Effect of okinawa propolis on PAK1 activity, Caenorhabditis elegans longevity, melanogenesis, and growth of cancer cells. J. Agric. Food Chem..

[101] Radu M, Semenova G, Kosoff R, Chernoff J (2014). PAK signalling during the development and progression of cancer. Nat. Rev. Cancer.

[102] Scherer D, Kumar R (2010). Genetics of pigmentation in skin cancer--a review. Mutat. Res..

[103] Fernandez LP (2009). Pigmentation-related genes and their implication in malignant melanoma susceptibility. Exp. Dermatol..

[104] Dentin R (2007). Insulin modulates gluconeogenesis by inhibition of the coactivator TORC2. Nature.

[105] Le Lay J (2009). CRTC2 (TORC2) contributes to the transcriptional response to fasting in the liver but is not required for the maintenance of glucose homeostasis. Cell Metab..

[106] Asati V, Mahapatra DK, Bharti SK (2017). K-Ras and its inhibitors towards personalized cancer treatment: pharmacological and structural perspectives. Eur. J. Med. Chem..

[107] Aboukameel A (2017). Novel p21-activated kinase 4 (PAK4) allosteric modulators overcome drug resistance and stemness in pancreatic ductal adenocarcinoma. Mol. Cancer Ther..

